# Simultaneous evaluation of abstinence and relapse using a Markov chain model in smokers enrolled in a two-year randomized trial

**DOI:** 10.1186/1471-2288-12-95

**Published:** 2012-07-07

**Authors:** Hung-Wen Yeh, Edward F Ellerbeck, Jonathan D Mahnken

**Affiliations:** 1 Department of Biostatistics, The University of Kansas Medical Center, Kansas City, Kansas, 66160; 2 Department of Preventive Medicine and Public Health, The University of Kansas Medical Center, Kansas City, Kansas, 66160

## Abstract

**Background:**

GEE and mixed models are powerful tools to compare treatment effects in longitudinal smoking cessation trials. However, they are not capable of assessing the relapse (from abstinent back to smoking) simultaneously with cessation, which can be studied by transition models.

**Methods:**

We apply a first-order Markov chain model to analyze the transition of smoking status measured every 6 months in a 2-year randomized smoking cessation trial, and to identify what factors are associated with the transition from smoking to abstinent and from abstinent to smoking. Missing values due to non-response are assumed non-ignorable and handled by the selection modeling approach.

**Results:**

Smokers receiving high-intensity disease management (HDM), of male gender, lower daily cigarette consumption, higher motivation and confidence to quit, and having serious attempts to quit were more likely to become abstinent (OR = 1.48, 1.66, 1.03, 1.15, 1.09 and 1.34, respectively) in the next 6 months. Among those who were abstinent, lower income and stronger nicotine dependence (OR = 1.72 for ≤ vs. > 40 K and OR = 1.75 for first cigarette ≤ vs. > 5 min) were more likely to have relapse in the next 6 months.

**Conclusions:**

Markov chain models allow investigation of dynamic smoking-abstinence behavior and suggest that relapse is influenced by different factors than cessation. The knowledge of treatments and covariates in transitions in both directions may provide guidance for designing more effective interventions on smoking cessation and relapse prevention.

**Trial Registration:**

clinicaltrials.gov identifier: NCT00440115

## Background

Smoking studies typically hypothesize intervention effects at a particular time point and analysis is performed in a cross-sectional manner using “time-naïve” approaches, e.g. the Pearson chi-square test or logistic regression. These approaches only use the outcome measures at the given time points, and appear to be appropriate when the research question focuses on these time point, or for short term smoking interventions with brief follow-up when smoking cessation is viewed as an acute, unidirectional problem (either smokers quit or they don’t). However, longitudinal studies often involve comparisons at multiple time points, and concern whether the intervention effects vary across time, which is beyond these “time-naïve” methods. Moreover, smoking cessation is increasingly being recognized as a dynamic process where people quit, relapse, and quit again, often with repeated cycles over years. Applying ‘time-naive’ approaches in these circumstances ignores this dynamic natural history of smoking cessation.

To address these concerns, researchers have proposed to use generalized estimating equations (GEE) and generalized linear mixed-effects models (GLMM) [1]. Both GEE and GLMM use repeated outcome measures and take the intra-personal association into account, and provide a means to comparing intervention effects at each time point and to examining whether the effects vary over time [2]. GEE provides population-averaged estimates, and the covariate effects can be interpreted as in standard logistic regression models. In contrast to GEE, GLMM is a subject-specific model. It estimates the probability that *an individual* would be abstinent (or smoking) at a given time point, allowing different propensity of abstinence (the random effects) among individuals. The two approaches also differ in other aspects, including the robustness or sensitivity to the assumptions of correlation structures and missing mechanisms [2]. Despite the differences, these two methods are inherently uni-directional, in the sense that they focus on the outcome measure of abstinence (or smoking) across time from smoking at baseline. For instance, suppose GEE suggests 10% of the participants are not smoking at time 1 and also 15% at time 2, which suggest an increase in abstinence rate. However, these numbers do not indicate whether the 10% subjects at time 1 continued abstinent until time 2 and another 5% smokers at time 1 stopped smoking at time 2, or all the 10% subjects at time 1 regressed to smoking at time 2 and another 15% smokers at time 1 became abstinent at time 2, or some of the 10% at time 1 remained abstinent into time 2 and some of the 90% smokers at time 1 turned abstinent at time 2. Similarly, suppose GLMM suggest an individual’s chance of abstinence is 10% at time 1 and 15% at time 2. This information does not tell how likely this individual would have relapse because relapse refers to a condition that an individual was previously abstinent. In other words, neither GLMM nor GEE is capable of modeling relapse and estimating this conditional probability of regression to smoking given abstinent at the previous time point.

On the other hand, a third method known as transition models provides a means to simultaneously investigate transitions in both directions from smoking to abstinent and from abstinent to smoking. Transition models provide the capability of identifying factors that might work differently in one direction versus the other. Through characterizing factors associated with cessation and those with relapse, we may be able to design more effective interventions for both smoking cessation and relapse prevention. Transition models are a family of models that characterize transition patterns in longitudinal studies. In these methods, the outcome measures are often countable states (e.g. smoking and abstinent can be the only two states that an observation can take). Models developed to handle outcomes observed at a set of scheduled time points are known as discrete-time models [3]. A common model is the discrete-time first-order Markov chain. It assumes that the future state will depend only on the current state but not the entire transition history, and transition probabilities from one state to another do not vary over time. With these two assumptions, we may summarize the transition of smoking status through the entire study window by a matrix with four probabilities: from smoking to smoking, from smoking to abstinent, from abstinent to abstinent, and from abstinent to smoking. Although the foundation of transition models is built on stochastic processes which tobacco researchers have seldom used, the implementation is straightforward when data are completely observed. It relies on counting the numbers of transitions from one state to another, and jointly models multiple logistic regression models, which most tobacco researchers are familiar with. See details in the Methods section.

In this article, we apply a non-homogeneous first-order Markov transition model to evaluate the transition of smoking behavior in a randomized smoking cessation trial *KanQuit*[4] in which smoking was addressed as a chronic illness and smokers underwent repeated interventions over a two year period of time. We also identify potential factors associated with transitions from smoking to not smoking (abstinence) and the factors associated with the other direction (relapse). We first perform the analysis using the available data alone. We consider the non-responses, particularly those due to lost-to-follow or consent withdraws, might be related to the actual smoking status. In other words, non-responses might be more likely to be smoking. For such situation, assuming non-responses to be missing at random is not proper and may introduce bias. Instead of coding all non-responses as smoking or non-smoking, we treat the missing as nonignorable and apply the selection modeling approach [5] following the recommendation of Hall et al. [2]. Parameter estimation is conducted by the expectation-maximization (EM) algorithm [6].

## Methods

### Data

Ellerbeck et al. [4] developed the *KanQuit* program, a 2-year randomized trial for smoking cessation delivered to rural smokers who consumed at least 10 cigarettes per day. All participants provided written informed consent. The Human Subjects Committee at The University of Kansas Medical Center approvedthe study (HSC# 9196). In this study, 750 adult participants, regardless of whether they were interested in stopping smoking, were recruited from rural primary care clinics across Kansas and randomized to one of three intervention groups: pharmacotherapy management alone (PM), pharmacotherapy management plus 1 – 2 counseling calls every 6 month (moderate-intensity disease management, or MDM), or pharmacotherapy management supplemented with up to 6 counseling calls every 6 month (high-intensity disease management, or HDM). The primary outcome, the self-reported 7-day abstinence (defined as not having smoked a cigarette during the previous 7 days), was recorded every 6 months from baseline to 24 months. As in many longitudinal studies, participants did not always respond and only 76% (552 individuals) responded at every time point.

Covariates obtained at enrollment included (1) demographic variables: age, gender, marital status, education level, annual household income, and the presence of children either ≤ 18 years or ≤ 6 years of age, (2) smoking characteristics: number of cigarettes smoked per day, nicotine dependence (time to first cigarette after waking up), (3) environmental smoking factors: number of friends that smoke, partner smoking status, whether anyone else smoked at home, and home smoking rules, and (4) psychosocial characteristics: global motivation to quit, confidence to quit, smoking self-efficacy questionnaire (SEQ) score, and whether they had made serious quit attempts in the past 6 months. Motivation and confidence to quit were assessed using 11-point (0 to 10) Likert scale measures, with greater scores indicating stronger motivation/confidence to quit smoking. The SEQ involves 12 items to assess participants’ confidence in their ability to refrain from smoking. These baseline characteristics are summarized in Table 1. Detailed information about the study design and covariates can be found in [4] and [7].

**Table 1:** Baseline characteristics of 750 study participants

**Characteristic**	**Total (n = 750)**	**PM (n = 250)**	**MDM (n = 249)**	**HDM (n = 251)**
Demographic				
Age, mean (SD)	47.2 (13.1)	47.0 (13.4)	48.2 (12.4)	46.4 (13.5)
Female, counts (%)	439 (58.5)	144 (57.6)	144 (57.8)	151 (60.2)
Marital: Married/partners (vs. others), counts (%)	504 (67.2)	170 (68.0)	167 (67.1)	167 (66.5)
Education: High school or less, counts (%)	385 (51.3)	128 (51.2)	129 (51.8)	128 (51.0)
Annual income ≤ 40 K, counts (%)*	453 (61.4)	155 (63.0)	151 (61.9)	147 (59.3)
Child under 18 (vs. no), counts (%)	294 (39.2)	97 (38.8)	89 (35.7)	108 (43.0)
Child under 6 (vs. no), counts (%)	106 (14.1)	33 (13.2)	31 (12.5)	42 (16.7)
Smoking				
Cigarettes smoked per day (CPD), mean (SD)	23.7 (10.4)	24.3 (11.0)	23.8 (10.3)	22.9 (10.0)
Nicotine dependence (time to first cigarette): within 5 minutes after waking up, counts (%)	285 (38.0)	93 (37.2)	103 (41.4)	89 (35.5)
Number of friends that smoked: < 3 friends (vs. ≥ 3 friends), counts (%)	289 (38.5)	99 (39.6)	95 (38.2)	95 (37.9)
Partner smoked? counts (%)	309 (41.2)	110 (44.0)	104 (41.7)	95 (37.9)
Other smokers at home? counts (%)	345 (46.0)	119 (47.6)	116 (46.6)	110 (43.8)
Home smoking rules				
Not allowed, counts (%)	204 (27.2)	65 (26.0)	67 (26.9)	72 (28.7)
Allowed some place, counts (%)	205 (27.3)	70 (28.0)	73 (29.3)	62 (24.7)
No rules (reference)				
Psychosocial				
Motivation to quit score (MOT), mean (SD)	8.6 (2.1)	8.7 (2.0)	8.6 (2.1)	8.6 (2.0)
Confidence to quit score (CON), mean (SD)	6.1 (2.7)	5.9 (2.7)	6.1 (2.8)	6.3 (2.6)
Smoking Self-Efficacy total score, mean (SD)	32.9 (10.7)	31.7 (10.5)	33.4 (11.0)	33.6 (10.5)
Serious quit attempt (SQA), counts (%)	180 (24.0)	56 (22.4)	60 (24.1)	64 (25.5)

* 12 participants did not respond.

### The Markov chain model

Consider a typical randomized trial in which participants are all smokers at enrollment (*t* = 0) and scheduled to have *T* follow-up visits with equal intervals after the treatment. Let $Y_{i,t}$ denote the smoking status, which takes value 1 if the status is smoking and 0 if abstinent, for the $i^{\mathrm{th}}$ individual at time t, i=1,2,…,N and t=0,1,…,T In a standard first-order Markov chain model, two assumptions are required: (1) the Markov property and (2) the stationary transition process. The Markov property refers to the probability that an individual is abstinent (or smoking) at a given time t depends only on her/his smoking status at the time t−1 but not on the entire history of observations, i.e., $Pr\left(Y_{i,t}\text{|}Y_{i,t-1},Y_{i,t-2},\ldots ,Y_{i,0}\right)=Pr\left(Y_{i,t}\text{|}Y_{i,t-1}\right)$ The assumption of stationary transition process refers to the assumption that the transition probabilities do not change over time, i.e. $Pr\left(Y_{i,t}=k\text{|}Y_{i,t-1}=j\right)=Pr\left(Y_{i,s}=k\text{|}Y_{i,s-1}=j\right)=\pi_{\mathrm{jk}}$ for any s≠t. Hence, the Markov chain model can be characterized by a transition matrix

(1) $$\prod_{i}=\left(\begin{array}{cc} \pi_{i,00} & \pi_{i,01}\\ \pi_{i,10} & \pi_{i,11} \end{array}\right)$$

where the first row provides probabilities of transitions from the state of abstinence at the previous time point whereas the second row shows transition probabilities from smoking; $\pi_{i,10}$ indicates the probability of becoming abstinent (given being smoking previously), and $\pi_{i,01}$ the chance of relapse. Here the subscript *i* indicates an allowance for patient-specific transition probabilities.

The assumption of stationary in time may not be realistic in practice. A variant model, known as time-inhomogeneous Markov chain, relaxes the stationary assumption and allows transition probabilities to vary across time, and can be denoted as $\pi_{i,jk}\left(t\right)$. 

To compare the crude treatment effects, one may compute the transition matrix for each treatment group, and then test equality of the matrices (see e.g., [8]) or use the regression method without covariates described below. When covariates are considered, one may model the transitions by logistic regression models

(2) $$\begin{array}{ll} \text{logit}\;\pi_{i,01}\left(t\right) & =\text{logit}\;\left[\text{Pr}\left(Y_{i,t}=1\text{|}Y_{i,t-1}=0\right)\right]\\ & =x_{i,t}^{'}\beta_{0}(1a) \end{array}$$

(3) $$\begin{array}{ll} \text{logit}\;\pi_{i,10}\left(t\right) & =\text{logit }\left[\text{Pr}\left(Y_{i,t}=0\text{|}Y_{i,t-1}=1\right)\right]\\ & =x_{i,t}^{'}\beta_{1}(1b) \end{array}$$

where $x_{i,t}$ is the vector from the design matrix for the *i*^th^ individual at time *t*, and (1a) and (1b) model relapse and abstinence of smoking, respectively. Note that covariates may have different effects in the two models, and $\beta_{0}$ and $\beta_{1}$ need not be equal. For ease of communication, we call (1a) and (1b) as Model (1) thereinafter unless one of them is specifically discussed.

### Statistical analysis

We first assume the missing mechanism is ignorable and analyze the data by Model (1) using the available data. Variables are selected based upon the Bayesian Information Criterion (BIC). We also consider the missing values to be outcome-dependent and not ignorable, and perform sensitivity analysis by selection modeling and jointly model the longitudinal smoking status and the missing process. Denote $R_{i,t}=1$ if $Y_{i,t}$ was observed and 0 otherwise. Because smoking-intervention trials typically enroll smokers only, their baseline smoking status is observed and $R_{i,0}=1$. A flexible model for missing mechanism [9] has the form

(4) $$\begin{array}{ll} \text{logit}\;\lambda_{i,t} & =\text{logit}\left[\text{Pr}\left(R_{i,t}=1\right)\right]\\ & =\alpha_{0}+\alpha_{1}r_{i,t-1}+\alpha_{2}y_{i,t-1}+\alpha_{3}y_{i,t}\\ & +x_{i,t}^{*}\alpha_{x} \end{array}$$

where $x_{i,t}^{*}$ can be any subset of $x_{i,t}$ and $\alpha_{x}$ is the associated parameter vector. Thus, $\alpha_{2}=\alpha_{3}=0$ implies missing complete at random (MCAR), $\alpha_{3}=0$ and $\alpha_{2}\neq 0$ indicate missing at random (MAR), and $\alpha_{3}\neq 0$ represents not missing at random (NMAR) [10].

The longitudinal smoking status (Models (1)) and the missing process (Model (2)) are then jointly modeled and estimated by the EM algorithm. Given all participants are smokers at enrollment, the complete data likelihood function $L\left(\theta ;Y,R\right)=\Pi_{i=1}^{N}L_{i}\left(\theta ;y_{i},r_{i}\right)$where $\left(\alpha ,\beta \right)$$=\left(\alpha_{0},\alpha_{1},\alpha_{2},\alpha_{3},\alpha_{x},\beta_{0},\beta_{1}\right)$, and the contribution of the $i^{\mathrm{th}}$ individual is

(5) $$\begin{array}{ll} L_{i}\left(\theta ;y_{i},r_{i}\right)= & \prod_{t=1}^{T}\left\{\lambda_{i,t},{}^{r_{i,t}},{\left(1-\lambda_{i,t}\right)}^{1-r_{i,t}}\right)\\ & \left(\prod_{j=0}^{1}\prod_{k=0}^{1}\pi_{i,jk}{\left(t\right)}^{I\left(y_{i,t-1}=j,y_{i,t}=k\right)}\right\} \end{array}$$

For ease of expression, we express $y_{i}=\left(y_{i}^{\mathrm{obs}},y_{i}^{\mathrm{mis}}\right)$ that respectively indicate observed and missing components. The E-step of the EM algorithm constructs the conditional expectation of the complete-data log-likelihood given the observed responses and the *v*-th iteration of the parameter estimates $Q\left(\theta ,\theta^{\left(\upsilon \right)}\right)=\sum_{i}^{N}Q_{i}\left(\theta ,\theta^{\left(\upsilon \right)}\right)$ where

(6) $$\begin{array}{ll} Q_{i}\left(\theta ,\theta^{\left(\upsilon \right)}\right)= & E_{Y_{i}^{\mathrm{mis}}}\left[\text{log}\;L_{i}\left(\theta ;y_{i},r_{i}\right)|X_{i},y_{i}^{\mathrm{obs}};\theta^{\left(\upsilon \right)}\right]\\ & =\sum_{y_{i}^{\mathrm{mis}}}\omega_{i}^{\left(\upsilon \right)}\text{log}\;L_{i}\left(\theta ;y_{i},r_{i}\right) \end{array}$$

and $\omega_{i}^{\left(\upsilon \right)}$ is the weight or the probability that the unobserved response has values $Y_{i}^{\mathrm{mis}}=y_{i}^{\mathrm{mis}}$ given the covariates and the parameter estimates at the *v*-th iteration. In the *M*-step, $Q\left(\theta ,\theta^{\left(\upsilon \right)}\right)$ is maximized with respect to θ by the Newton–Raphson algorithm. In practice, we start with initial values of $\theta^{\left(0\right)}$ to construct the conditional expectation $Q\left(\theta ,\theta^{\left(0\right)}\right)$ which is maximized with respect to parameters to update θ ($\theta^{\left(1\right)}$), and then repeat the E- and the M-steps iteratively until the parameter estimates converge. The standard error (SE) of parameter estimates are estimated by non-parametric bootstrapping [11] with 1,000 samples.

Analysis was performed on SAS version 9.2. PROC NLMIXED was used for analysis on available data. PROC IML was applied to conduct the selection modeling estimated by the EM algorithm, and a SAS macro was developed for bootstrapping (Additional file 1: Appendix).

## Results

### Using available data alone (ignore missing mechanism)

**Figure 1:**
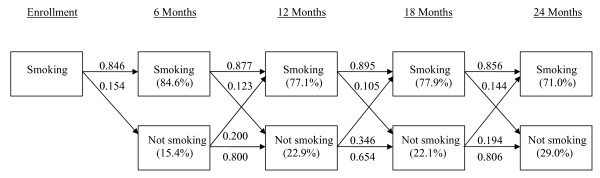
Transition probabilities of self-reported smoking status based on the available observations.

If the assessment of smoking status was complete for all of the 750 participants, there should have been 750 x 4 = 3000 observations. Nonetheless, these patients provided 2540 observations, or around 15.3% missing values. Figure 1 describes the transition of self-reported smoking status based on *available* observations for all the 750 participants. At Month 6, 15.4% of participants became abstinent. Among these participants who also self-reported their status at Month 12, 20% regressed to smoking; among those continued smoking at Month 6 and self-reported at Month 12, 12.3% turned to abstinent. Transition probabilities between the other time points are interpreted in the similar manner. The proportions of smoking and not smoking at each time point (based on available data) are shown in parentheses within each box. The group-specific probabilities can be found in Table 3 of [4]. Results of using available data alone in the Markov model suggest that the crude intervention effects were significantly different across treatment arms in promoting smoking abstinence ($F_{2,2540}$= 5.16, p = 0.006), but not in preventing relapse ($F_{2,2540}$= 1.72, p = 0.179). When covariates were considered in the model, some covariates were also significantly associated with transitions from smoking to abstinence and some other covariates from abstinent to smoking:

#### Model (1a) Transition from smoking to abstinent

Given smoking currently, the odds in favor of abstinence *in the next 6 month* was higher among male smokers than females (OR = $e^{0.504}$ = 1.66, p = 0.0002, Table 2), among smokers consuming fewer cigarettes per day at baseline (OR = $e^{-(-0.033)}$ = 1.03, p < 0.0001), among those with higher motivation and confidence (OR = $e^{0.144}$ = 1.15, p < 0.0001 and OR = $e^{0.083}$ = 1.09, p = 0.0014, respectively), and among those who had serious attempt to quit (OR = $e^{0.295}$ = 1.34, p = 0.043). After adjusting for these covariates, the overall treatment effects were significant ($F_{2,2536}$= 3.65, p = 0.026. Note that the degrees of freedom are 2536 due to 4 additional observations without income information). Specifically, the odds in favor of abstinence in the next 6 month was significantly higher in the HDM arm as compared to the control (PM) group (OR = $e^{0.392}$ = 1.48, p = 0.013) and in MDM vs. HDM (OR = $e^{0.392-0.056}$ = 1.40, p = 0.037) at 0.05 significance level, but not between MDM and PM (OR = $e^{0.056}$ = 1.06, p = 0.74). The temporal variation in transition to abstinence was not significant ($e^{0.056}$ = 1.63, p = 0.180, results not shown).

**Table 2:** Transition model estimates of abstinence and relapse to smoking

	**Available data only§**			**Selection modeling 1**			**Selection modeling 2**		
	**Estimate**	**SE**	**p-value**	**Estimate**	**SE**	**p-value**	**Estimate**	**SE**	**p-value**
Transition model									
From smoking to abstinent (abstinence model)									
Intercept	-3.348	0.456	< 0.0001	-3.381	0.503	< 0.0001	-3.382	0.502	< 0.0001
MDM	0.056	0.166	0.737†	-0.026	0.193	0.446	-0.022	0.188	0.453
HDM	0.392	0.158	0.013†	0.322	0.177	0.034	0.329	0.171	0.027
Gender	0.504	0.137	0.0002	0.461	0.158	0.002	0.462	0.157	0.002
Cigarettes per day	-0.033	0.008	< 0.0001	-0.035	0.009	< 0.0001	-0.035	0.009	< 0.0001
Motivation to quit	0.144	0.040	0.0004	0.150	0.044	0.0003	0.150	0.044	0.0003
Confidence to quit	0.083	0.026	0.0014	0.083	0.028	0.002	0.083	0.028	0.002
Serious quit attempt	0.295	0.146	0.043	0.333	0.164	0.021	0.332	0.164	0.021
From abstinent to smoking (relapse model)									
Intercept	-0.939	0.338	0.006	-0.979	0.376	0.005	-0.977	0.377	0.005
Month 18	0.763	0.318	0.016*	0.792	0.306	0.005	0.788	0.301	0.004
Month 24	-0.029	0.342	0.933*	-0.012	0.331	0.486	-0.009	0.332	0.489
MDM	-0.708	0.331	0.033‡	-0.624	0.372	0.047	-0.629	0.375	0.047
HDM	-0.409	0.305	0.181‡	-0.366	0.361	0.155	-0.371	0.363	0.153
Income (> 40 K vs. ≤ 40 K)	-0.564	0.259	0.029	-0.575	0.308	0.031	-0.576	0.307	0.030
First cigarette (≤ vs. > 5 min)	0.588	0.269	0.029	0.600	0.322	0.031	0.601	0.322	0.031
Missing model									
Intercept	—	—	—	-0.527	0.339	0.060	-0.626	0.432	0.074
Month 12	—	—	—	—	—	—	0.366	0.196	0.031
Month 18	—	—	—	—	—	—	0.072	0.176	0.341
Month 24	—	—	—	—	—	—	0.597	0.193	0.001
MDM	—	—	—	—	—	—	-0.416	0.164	0.006
HDM	—	—	—	—	—	—	-0.496	0.173	0.002
$R_{t-1}$	—	—	—	3.240	0.191	< 0.0001	3.367	0.194	< 0.0001
$Y_{t-1}$	—	—	—	0.013	0.319	0.484	0.067	0.287	0.408
$Y_{t}$	—	—	—	-0.428	0.532	0.211	-0.402	0.443	0.182

§ -2 log-L = 1944.4; AIC = 1974.4; BIC = 2062.0;

† Overall treatment effects ($F_{2,2536}$= 3.65, p = 0.026); * time ($F_{2,2536}$= 4.69, p = 0.009); ‡ overall treatment ($F_{2,2536}$= 2.31, p = 0.099).

#### Model (1b) Transition from abstinent to smoking

Among the participants not currently smoking, those with lower income (OR = $e^{-(-0.564)}$ = 1.76, p = 0.029) and stronger nicotine dependence (i.e. first cigarette within 5 minutes after waking up) at baseline were more likely to have relapse (OR = $e^{0.588}$ = 1.80, p = 0.029) in the next 6 month as compared to their counterparts. Note the significant time effects ($F_{2,2536}$ = 4.89, p = 0.008), indicating that the relapse rates changed over time (Figure 1). After adjusting for temporal variation and income as well as nicotine dependence, the overall effects on preventing relapse were still not significant ($F_{2,2536}$ = 2.30, p = 0.100) though relapse seemed to be less likely in the MDM group as compared to the PM group (OR = $e^{-0.763}$ = 0.49, p = 0.033).

### Sensitivity analysis

In sensitivity analysis, we consider a couple of possible missing mechanisms and present only two of them in Table 2. In one analysis (Selection modeling 1), we assume missingness depends on whether the smoking status is observed at the previous time point ($R_{t-1}$), the actual smoking status at previous ($Y_{t-1}$) and current time point ($Y_{t}$); in the other analysis, we further consider time and treatment arms in the missing model. In either case, the conclusion of significance remains the same.

The missing mechanism model should be interpreted with caution. Apparently, missingness significantly depends on *R*_*t*-1_ (p < 0.0001) and treatment arms (p = 0.006 and 0.002 for the MDM vs. PM and HDM vs. PM, respectively), but not on *Y*_*t*-1_ or *Y*_*t*_ in either sensitivity analysis, so the missing mechanism seems to be MCAR. However, when the missing process is jointly modeled with the repeated outcome measures, various parameter values may come to the same likelihood. In other words, even if the transition and the missing models are correctly specified, joint modeling can reduce bias in parameter estimates of the transition model, but may not do so in those of the missing model. The missing model parameters may take a wide range of values and the estimates may be biased. This issue is known as the “identifiability” problem (see e.g. [12] and [13]). Hence, some literature only reports the results for the main outcome model but not the missing model (see e.g. [13] and [14]).

## Discussion

In this work, we applied a Markov chain model to study the transitions from smoking to abstinence and from abstinence to smoking among the 750 patients in the *KanQuit* trial. Different factors associated with each type of transition were identified: gender, the baseline daily cigarettes consumption, the baseline motivation and confidence to quit, as well as having serious attempt to quit were associated with transitions from smoking to abstinent, whereas income and nicotine dependence were associated with relapse. The intervention effects were significant in promoting abstinence but not so in preventing relapse (in spite of the MDM group showing a trend of a lower relapse rate), which is probably due to the fact that too few observations of such transitions were available. The sensitivity analyses confirmed the conclusion.

Cox et al. [15] studied the predictors of smoking abstinence at Month 6 and Month 24, separately, using the 592 participants who completed the assessment at both time points. They identified male gender and lower baseline daily cigarettes consumption. They did not find intervention effects significant at either time point. Ellerbeck et al. [4] applied the GLMM to evaluate an individual’s odds in favor of abstinence in treatment arms (without covariates), with and without imputation (single imputation of coding all missing to be smoking and not smoking, respectively), and found a significantly higher odds in the HDM group than PM, but not between MDM and PM, or between the two intervention groups, except when all missing were coded as smoking. These findings seem to be consistent with the current work (the portion of transitions from smoking to abstinent or Model (1a)), but the interpretations are different. The GLMM (specifically the random intercept model) suggests that the odds in favor of abstinence for *an individual* in the HDM group was higher than the odds if *the same individual* were in the PM group, assuming the intervention effects were the same for all individuals. On the other hand, the transition model indicates that (1) among participants who were smoking at a given time point, the odds in favor of abstinence *in the next 6-month* was higher in the HDM than the PM group, in males than females, in those with higher motivation and confidence to quit, and in those having serious attempts to quit; and (2) among participants who were abstinent at a given time point, the odds of relapse *in the next 6-month* was greater at Month 18 (see Figure 1), and among those with lower income and stronger nicotine dependence. Note that relapse refers to turning from abstinence to smoking, thus analysis of relapse must confine to information of participants who ever quitted at some time point(s).

As mentioned in Introduction, GEE and GLMM may estimate the population abstinence rate or an individual’s chance of not smoking, respectively, but they do not provide the relapse rates which are conditional probabilities. Note that subtracting the abstinence rate or an individual’s chance of not smoking from 100% does not give the relapse rate because this number can include participants who ever quitted smoking and those who never. If a smoker continued smoking without abstinence until the observed time, we wouldn’t consider this observation as relapse. This limitation in GEE or GLMM is a strength of transition models. Another strength of Markov models is, when the outcome is multinomial, Markov models may estimate parameters more accurately and provide greater power in rejecting the null hypothesis than GEE [16].

Moreover, the transition models may serve as an intervention diagnostic tool, which can be particularly beneficiary for studies failing to show significant intervention effects in abstinence rates. An intervention may fail because either it does not motivate smokers to stop smoking, or it does motivate abstinence but fails to prevent relapse. This question can be answered by the transition patterns shown in Figure 1 and Markov models. Researchers may examine the factors associated with the transitions in both directions, and modify the intervention by incorporating these factors. In the *KanQuit* example, the results suggest that on the basis of the current disease management interventions, the intervention effects may be further improved by enhancing smokers’ motivation and confidence to quit and quit attempts, as well as reducing baseline daily cigarette consumption and/or nicotine dependence.

In the sensitivity analysis (Table 2), we note that the current and previous smoking status are not significant in the missing models. However, it is still premature to claim that missing was ignorable. In fact, even though missing is non-ignorable, the impact of missing values may be mild to moderate in some occasions. When observations are strongly correlated, we may borrow the information from the observed values at the neighboring time points to predict the missing values, and the bias due to the missing values can be reduced ([17], Section 2.5). Similarly, for the Markov chain models, a strong association among the repeated measures or strong dependence of current observations on previous observations may reduce bias in estimation due to non-ignorable missing [18]. In this study, high probabilities of staying in a current state (from smoking to smoking and from abstinent to abstinent in Figure 1) suggest strong dependence of current states on the previous ones, which helped reduce the impact of non-ignorable non-responses.

As mentioned earlier, non-responses can be caused by various reasons and the true missing mechanism cannot be determined by the data at hand. If ignorable missing can be justified, an alternative approaches to handle missing values are to apply multiple imputation [17,19] or the multi-step transition probabilities [8]. The probabilities of transitions between two consecutive time points, as described in the Method section, are called the one-step probabilities. When missing values exist, one should consider all possible states for the missing values and sum up the one-step probabilities for all possible routes to obtain the multi-step transition probabilities. This approach may reduce SE and increase statistical power as the EM algorithm [20].

Another issue is about the validity of self-report smoking status. Ellerbeck et al. [4] showed deviation in abstinence rates between the self-reported status and the validated saliva cotinine levels (threshold of 15 ng/mL) among the 58% participants who provided saliva samples. When measurements are subject to errors, data are often analyzed by latent variable methods such as hidden Markov models [18] or latent transition analysis (LTA) [21-23]. We also attempted to fit the *KanQuit* data by the LTA [24]. However, the LTA model did not seem to fit the data better than the conventional Markov model, and the results are contradictory to our understanding (nearly no chance of relapse and participants would have 14.8% chance of incorrectly reporting they were smoking when they were actually not). Therefore, the results are not presented in this work.

## Conclusions

In this article, we discuss and demonstrate how a Markov chain model may use the information in randomized smoking cessation trials that the GEE or mixed-effects models do not utilize, and provide additional findings. With the Markov chain model, we are able to learn about the factors associated with relapse to smoking among those who are temporarily abstinent as well as the factors associated with abstinence. Therefore, to fully investigate longitudinal smoking cessation randomized trials, we encourage researchers to apply transition models together with either GEE or GLMM. GEE or GLMM compares treatment effects between intervention groups and examines the temporal profiles of the treatment effects; Markov models provide knowledge about transitions between abstinence and relapse in both directions. This knowledge may provide guidance in evaluating and designing more effective interventions for smoking cessation and relapse prevention. When non-response causes considerable missing values (e.g. 10% or more) and if non-ignorable missing is considered, sensitivity analysis based on a couple of missing mechanisms should be examined. The results of the missing model should be interpreted with caution.

## Competing interests

The authors declare that they have no competing interests.

## Authors' contributions

HY: performed statistical analysis, interpreted results, and drafted the manuscript. EFE: designed the clinical trial, procured funding for the study, directed the implementation of the trial, and revised the draft. JDM: provided data management oversight and statistical analysis for the *KanQuit* trial, assisted in the developing the analysis plan for the current analysis, and revised the draft. All three authors read and approved the final manuscript.

## Pre-publication history

The pre-publication history for this paper can be accessed here:

http://www.biomedcentral.com/1471-2288/12/95/prepub

## Supplementary Material

Additional file 1 **Appendix.** SAS codes. Click here for file
