# Supplementary material for: Simultaneous evaluation of abstinence and relapse using a Markov chain model in smokers enrolled in a two-year randomized trial
Source: BMC Med Res Methodol. 2012 Jul 7;12:95. doi: 10.1186/1471-2288-12-95 (PMC3599722; doi:10.1186/1471-2288-12-95)
Supplement: Additional file 1 — Appendix. SAS codes. [file 1471-2288-12-95-S1.docx]

**Appendix SAS codes**

*********************************************************

***** Data: y_long (long format for PROC NLMIXED)

***** y_wide (wide format for EM)

*********************************************************;

title "available data only";

**proc** **nlmixed** data=y_long;

parms b0=**0** b1=**0** b2=**0** b3=**0** b4=**0** b5=**0** b6=**0** b7=**0** /*abstinence coeff */

a0_1=**0** a0_2=**0** a0_3=**0** a1=**0** a2=**0** a3=**0** a4=**0**; /*relapse coeff */

logit1 = b0 + b1*MDM + b2*HDM + b3*male + b4*cignum + b5*mot + b6*con + b7*sqa;

logit0 = a0_1 + a0_2*t2 + a0_3*t3 + a1*MDM + a2*HDM + a3*income + a4*Fcig5min;

/* t2 and t3: dummy variables for Months 18 and 24 */

p0 = **1**/(**1** + exp(-logit0)); /* p0: prob of abstinence */

p1 = **1**/(**1** + exp(-logit1)); /* p1: prob of relapse */

if y_1 ^= **.** and y ^=**.** then do; /* y_1: smoking status at previous time point */

/* y: smoking status at time t */

if y_1 = **0** then ll = y*log(p0) + (**1** - y)*log(**1** - p0);

else if y_1 = **1** then ll = (**1** - y)*log(p1) + y*log(**1** - p1);

end;

else if y_1 = **.** or y = **.** then ll = **0**;

model y ~ general(ll);

contrast "arm in abstinence" b1, b2;

contrast "arm in relapse" a1, a2;

contrast "time in relapse" a0_2, a0_3;

**run**;

* create all possible outcomes for missing values in selection modeling;

**%macro** ***loop4***;

if y4 ^= **.** then do; /* y1 - y4: smoking status at time 1 to time 4 */

s4 = y4; output;

end;

else do;

s4 = **0**; output;

s4 = **1**; output;

end;

**%mend**;

**%macro** ***loop3***;

if y3 ^= **.** then do;

s3 = y3;

%***loop4***;

end;

else do;

s3 = **0**;

%***loop4***;

s3 = **1**;

%***loop4***;

end;

**%mend**;

**%macro** ***loop2***;

if y2 ^= **.** then do;

s2 = y2;

%***loop3***;

end;

else do;

s2 = **0**;

%***loop3***;

s2 = **1**;

%***loop3***;

end;

**%mend**;

**%macro** ***loops***;

data temp4;

set y_wide;

drop y0 - y4; s0=**1**;

if y1 ^= **.** then do;

s1 = y1;

%***loop2***;

end;

else do;

s1 = **0**;

%***loop2***;

s1 = **1**;

%***loop2***;

end;

run;

**%mend**;

%***loops***;

**%macro** ***EM***;

data temp5; set temp4; seq = _n_; drop r0 - r4 s0 - s4; one = **1**;

t1=**0**; t2=**0**; t3=**0**; r_1 = r0; r = r1; s_1 = s0; s = s1; output;

t1=**1**; t2=**0**; t3=**0**; r_1 = r1; r = r2; s_1 = s1; s = s2; output;

t1=**0**; t2=**1**; t3=**0**; r_1 = r2; r = r3; s_1 = s2; s = s3; output;

t1=**0**; t2=**0**; t3=**1**; r_1 = r3; r = r4; s_1 = s3; s = s4; output;

run;

proc iml;

start binest;

a=repeat(**0**,nrow_a,**1**); /* initial values for parm. in the missing model */

b=repeat(**0**,nrow_b,**1**); /* initial values for parm. in the abstinence model*/

c=repeat(**0**,nrow_c,**1**); /* initial values for parm. in the relapse model */

p_lik = repeat(**.**,rows_x,**4**);

one = j(rows_x,**1**,**1**); /* create a vector of 1's for the design matrix */

dif_max = **1**;

do iter = **1** to **100** while(dif_max > **1e-3**);

old_a = a; old_b = b; old_c = c;

run pr_lik;

run weight;

run newraph;

trans_a = t(a);

trans_b = t(b);

trans_c = t(c);

diff_a = a - old_a;

diff_b = b - old_b;

diff_c = c - old_c;

dif_max = max(diff_a,diff_b,diff_c);

end;

finish;

start pr_lik; /* compute the prob. for constructing likelihood function */

logit_r = xa*a; logit_b = xb*b; logit_c = xc*c;

p_lik[,**1**] = **1**/(**1** + exp(-logit_r)); /* Pr(r=1) */

p_lik[,**2**] = **1**/(**1** + exp(-logit_b)); /* Pr(y(t)=0|y(t-1)=1) */

p_lik[,**3**] = **1**/(**1** + exp(-logit_c)); /* Pr(y(t)=1|y(t-1)=0) */

/* expetced likelihood evaluated at v-th iterate */

p_lik[,**4**] =

(p_lik[,**1**] ## r) # ((**1** - p_lik[,**1**]) ## (**1** - r)) #

( s_1 # ( (p_lik[,**2**] ## (**1** - s)) # ((**1** - p_lik[,**2**]) ## s ) ) +

(**1** - s_1) # ( (p_lik[,**3**] ## s ) # ((**1** - p_lik[,**3**]) ## (**1** - s)) ));

finish;

start weight; /* compute the weights in the Q function */

lik1 = (id_seq[,**2**])||(p_lik[,**4**]);

unique_seq = uniqueby(lik1,**1**,**1**:rows_x); prod_seq = repeat(**.**,rows_x,**1**);

do i = **1** to nrow(unique_seq);

if i=nrow(unique_seq) then index1=unique_seq[i]:rows_x;

else index1=unique_seq[i]:unique_seq[i+**1**]-**1**;

submat1 = lik1[index1,**2**];

nrows_sub1 = nrow(submat1);

prod = **1**;

do ii = **1** to nrows_sub1;

prod = prod*submat1[ii];

end;

prod_sub = repeat(prod,nrow(submat1),**1**);

prod_seq[index1] = prod_sub;

end;

lik2 = (id_seq[,**1**])||prod_seq;

unique_id = uniqueby(lik2,**1**,**1**:rows_x); sum_id = repeat(**.**,rows_x,**1**);

do j = **1** to nrow(unique_id);

if j=nrow(unique_id) then index2=unique_id[j]:rows_x;

else index2=unique_id[j]:unique_id[j+**1**]-**1**;

submat2 = lik2[index2,**2**];

sum2 = repeat(sum(submat2)/no_trans,nrow(submat2),**1**);

sum_id[index2] = sum2;

end;

wt = prod_seq/sum_id;

finish;

start newraph; /* newton-raphson algorithm */

Q = sum(wt # (

r # log(p_lik[,**1**]) + (**1** - r) # log(**1** - p_lik[,**1**]) +

s_1 # ( (**1** - s) # log(p_lik[,**2**]) + s # log(**1** - p_lik[,**2**]) ) +

(**1** - s_1) # ( s # log(p_lik[,**3**]) + (**1** - s) # log(**1** - p_lik[,**3**]) )

));

u_a = t(xa)* (( r - p_lik[,**1**]) # wt);

u_b = t(xb)* ( s_1 # ((**1** - s) - p_lik[,**2**]) # wt);

u_c = t(xc)* ( (**1** - s_1) # ( s - p_lik[,**3**]) # wt);

/* partition into parts to facilitate computation */

dividend = **1000**; folds = int(rows_x/dividend);

n_row_a = nrow(a); n_row_b = nrow(b); n_row_c = nrow(c);

H_a = j(n_row_a,n_row_a,**0**);

H_b = j(n_row_b,n_row_b,**0**);

H_c = j(n_row_c,n_row_c,**0**);

do subset = **1** to folds;

lb = (subset - **1**)*dividend + **1**; ub = dividend*subset;

xa_sub = xa[lb:ub,];

xb_sub = xb[lb:ub,];

xc_sub = xc[lb:ub,];

p_lik_sub = p_lik[lb:ub,];

wt_sub = wt[lb:ub];

D_sub_a = diag(p_lik_sub[,**1**] # (**1** - p_lik_sub[,**1**]) # wt_sub);

D_sub_b = diag(p_lik_sub[,**2**] # (**1** - p_lik_sub[,**2**]) # wt_sub);

D_sub_c = diag(p_lik_sub[,**3**] # (**1** - p_lik_sub[,**3**]) # wt_sub);

H_a = H_a - t(xa_sub)* D_sub_a * xa_sub;

H_b = H_b - t(xb_sub)* D_sub_b * xb_sub;

H_c = H_c - t(xc_sub)* D_sub_c * xc_sub;

end;

if mod(rows_x,dividend) = **0** then do;

H_a = H_a;

H_b = H_b;

H_c = H_c;

end;

else if mod(rows_x,dividend) > **0** then do;

last_lb = dividend*folds + **1**;

xa_last = xa[last_lb:rows_x,];

xb_last = xb[last_lb:rows_x,];

xc_last = xc[last_lb:rows_x,];

wt_last = wt[last_lb:rows_x];

p_lik_last = p_lik[last_lb:rows_x,];

D_last_a = diag(p_lik_last[,**1**] # (**1** - p_lik_last[,**1**]) # wt_last);

D_last_b = diag(p_lik_last[,**2**] # (**1** - p_lik_last[,**2**]) # wt_last);

D_last_c = diag(p_lik_last[,**3**] # (**1** - p_lik_last[,**3**]) # wt_last);

H_a = H_a - t(xa_last)* D_last_a * xa_last;

H_b = H_b - t(xb_last)* D_last_b * xb_last;

H_c = H_c - t(xc_last)* D_last_c * xc_last;

end;

cova = -inv(H_a); covb = -inv(H_b); covc = -inv(H_c);

a = a + cova*u_a;

b = b + covb*u_b;

c = c + covc*u_c;

finish;

**%mend**;

**%macro** ***read_in***;

use y_wide;

read all var{sid2} into sid2; /* sid2: study id */

n_subject = nrow(sid2);

use temp5; read all var{sid2 seq} into id_seq;

use temp5; read all var{r} into r;

use temp5; read all var{s} into s;

use temp5; read all var{s_1} into s_1;

no_trans = **4**; * no. of transitions;

rows_x = nrow(id_seq);

nrow_a = ncol(xa);

nrow_b = ncol(xb);

nrow_c = ncol(xc);

**%mend**;

**%macro** ***joint_mis***; /* obtain point est. in EM */

proc iml;

%***EM***;

use temp5;

read all var{one t1 t2 t3 mdm hdm r_1 s_1 s} into xa;

/* one is a vector of 1's;

r_1 indicates whether outcome was observed at previous time pt.;

s_1 is the (observed or the potential value if missing) outcome at t-1;

s is the (observed or the potential value if missing) outcome at t-1;

*/

use temp5;

read all var{one mdm hdm male CigNum mot con sqa} into xb;

/* design matrix for the abstinence model */

read all var{one t2 t3 mdm hdm income Fcig5min} into xc;

/* design matrix for the relapse model */

%***read_in***;

run binest;

name_a = {intercept, t1, t2, t3, mdm, hdm, r_1, s_1, s};

name_b = {intercept, mdm, hdm, male, CigNum, mot, con, sqa};

name_c = {intercept, t2, t3, mdm, hdm, income, Fcig5min};

par_name = name_b//name_c//name_a;

title "missing ~ month arm r(t-1) y(t-1) y(t)";

print Q iter, name_b b, name_c c, name_a a;

quit;

**%mend**;

%***joint_mis***;

/* Bootstrapping to estimate variance in EM */

**%macro** ***seq***;

data temp4;

set boot_i;

drop y0 - y4; s0=**1**;

if y1 ^= **.** then do;

s1 = y1;

%***loop2***;

end;

else do;

s1 = **0**;

%***loop2***;

s1 = **1**;

%***loop2***;

end;

run;

**%mend**;

**%macro** bootstrap(boot); /* boot: number of bootstrap samples */

data arm1 (where=(arm=**1**))

arm2 (where=(arm=**2**))

arm3 (where=(arm=**3**));

set y_wide;

run;

data boot;

%do grp = **1** %to **3**;

do sample = **1** to &boot;

do i = **1** to nobs;

pt = round(ranuni(&grp)*nobs);

set arm&grp nobs = nobs point=pt;

output;

end;

end;

%end;

stop;

run;

data theta;

sample = **0**; iter=**0**; Q = **.**;

b0=**0**; b1=**0**; b2=**0**; b3=**0**; b4=**0**; b5=**0**; b6=**0**; b7=**0**;

c0=**0**; c0_1=**0**; c0_2=**0**; c1=**0**; c2=**0**; c3=**0**; c4=**0**;

a0_0=**0**; a0_1=**0**; a0_2=**0**; a0_3=**0**; a1=**0**; a2=**0**; a3=**0**; a4=**0**; a5=**0**;

run;

%do sample = **1** %to &boot;

data boot_i; set boot; if sample = &sample; sid2 = _n_; run;

%***seq***;

%***em***;

proc append data=theta_v base=theta force; run;

%end;

**%mend**;

%***bootstrap***(**1000**);

**data** theta; set theta; if sample > **0**; **run**;

title "SE by bootstrapping";

**proc** **means** data=theta n std; **run**;
